# Outcomes of Outpatient Versus Inpatient Induction of Labor: A Systematic Review and Meta-Analysis

**DOI:** 10.7759/cureus.69535

**Published:** 2024-09-16

**Authors:** Mohammed Mustafa, Mohamed Babiker, Fatema Abusin, Tamador Mohammed, Tarig Awadalla

**Affiliations:** 1 Obstetrics and Gynaecology, Ahfad University For Women, Khartoum, SDN; 2 Obstetrics and Gynaecology, University Hospital Galway, Galway, IRL; 3 Obstetrics and Gynaecology, Ahfad University For Women, School of Medicine, Khartoum, SDN; 4 Obstetrics and Gynaecology, Nottingham University Hospital, Nottingham, GBR; 5 Obstetrics and Gynaecology, Mayo University Hospital, Castlebar, IRL

**Keywords:** outpaitent induction of labour, home induction of labour, foley catheter induction, home mechincal induction, hormonal induction at home

## Abstract

Induction of labor (IOL) has become a common practice in obstetrics, leading to an increase in antenatal admissions and workload. This review aims to explore the available options for outpatient IOL and their effectiveness. We conducted an electronic search for trials on Cochrane, PubMed, Google Scholar, and Web of Science databases for randomized control trials (RCTs) comparing inpatient and outpatient labor induction and covering the period until 2024. We selected randomized trials that compared IOL in outpatient vs. inpatient settings and involved mechanical or hormonal agents. The participants were pregnant women with singleton fetuses who were more than 37 weeks and low risk for IOL with a Bishop score <6.

When comparing outpatient and inpatient induction methods, we found no significant differences in cesarean section rates and vaginal delivery. Outpatient induction generally resulted in shorter hospital stays. Using a Foley catheter for outpatient induction reduced the cesarean section rate and total hospital stay. There were no safety concerns with this approach. IOL in this analysis was shown to be similar to inpatient IOL in most of the measured outcomes. Implementation of IOL in an outpatient setting proved to be safe with similar outcomes to inpatient IOL.

## Introduction and background

Induction of labor (IOL) is a routine obstetric practice that currently places a significant workload on obstetric units, especially with the rising number of inductions. Other studies in the literature have tested the concept of outpatient IOL as a method that may reduce this burden and increase satisfaction among women and staff without compromising patient safety or obstetric and neonatal outcomes [1].

In outpatient settings, labor induction may involve the use of mechanical devices such as a single or double balloon Foley catheter, osmotic Hegar's dilators like Dilapan-S®, or hormonal techniques such as prostaglandin gel or inserts, mifepristone, and misoprostol. Most modern obstetric units that use mechanical methods and perform labor induction in an outpatient setting typically work with low-risk patients and use these methods primarily when the overall clinical risk is low. Prostaglandin inserts, on the other hand, have been the subject of multiple studies and are beneficial for outpatient IOL, particularly in high-income countries where prompt follow-ups and interventions are guaranteed.

To investigate the efficacy and safety of outpatient induction compared to inpatient IOL, we compiled all relevant randomized controlled trials (RCTs) for a meta-analysis and performed a subgroup analysis comparing outpatient versus inpatient induction using a Foley catheter. Our updated review aims to endorse decisions regarding the use of outpatient IOL by examining the effects of mechanical and hormonal agents and general obstetric and neonatal outcomes.

## Review

Methods

This meta-analysis was conducted as per the Preferred Reporting Items for Systematic Reviews and Meta-Analyses (PRISMA) guidelines and the protocol for systematic reviews and meta-analyses in 2020. There were no major amendments made to the protocol, except for the addition of a single secondary outcome, which was the total duration of oxytocin use. This trial was prospectively registered with PROSPERO [CRD42024538570].

Inclusion and Exclusion Criteria

We rigorously focused on RCTs comparing outpatient and inpatient IOL. The exclusion criteria were as follows: inpatient RCTs without outpatient comparison, induction of high-risk pregnant population, and outpatient induction with other methods that are still under research like Isosorbide mononitrate, and non-RCT designs.

Overall, we included 18 RCTs, all conducted in high-income countries, with 4886 women enrolled and randomized for outpatient IOL versus inpatient IOL as follows. Nine studies examined outpatient IOL by Foley catheter versus inpatient IOL by Foley catheter: Beckmann et al. [2]; Elizabeth et al. [3]; Haavisto et al. [4]; Hamdan et al. [5]; Kuper et al. [6]; Policiano et al. [7]; Wilkinson et al. [8]; Sciscione et al. [9]; and Rahman et al. [10]. Three studies compared outpatient and inpatient IOL with prostaglandin (Dinoprostone gel/insert); Biem et al. [11]; Wilkinson et al. [12]; and Ryan et al. [13]. Two studies compared outpatient IOL by Foley catheter versus inpatient IOL by prostaglandin: Henry et al. [14], and Wise et al. [15]. One study compared outpatient IOL by Hegar's dilator Dilapan-S® and Foley catheter: Lu et al. [16]. One study compared outpatient IOL with vaginal misoprostol versus an inpatient placebo: Stitely et al. [17]. One study compared outpatient IOL with mifepristone versus inpatient IOL with mifepristone: Baev et al. [18]. One study compared outpatient and inpatient IOL by hygroscopic osmotic dilator: Saad et al. [19].

There were six studies from the USA, four from Australia, four from Canada and Malaysia, and one study each from Finland, New Zealand, Russia, and Portugal. All studies reported their source of funds, which was not commercial; however, Saad et al. [19], reported receiving funds from Medicem company, which is the supplier of hygroscopic dilator Dilapan-S®, though the authors declared no conflict of interest. Also, all studies declared no conflict of interest except for Sciscione et al. [9], Ryan et al. [13], and Stitely et al. [17], who did not comment on it.

Outcomes extracted from studies included broad clinical obstetric outcomes, primarily cesarean section rate, operative vaginal delivery, and vaginal delivery rate. Neonatal outcomes mainly focused on special care baby unit (SCBU) admission. Secondary outcomes include SCBU admission, maternal satisfaction, induction to amniotomy Interval, Induction to delivery Interval, use of oxytocin, intrapartum fever, induction to spontaneous rupture of membrane, tachysystole, operative vaginal delivery, total hospital stay, and vaginal delivery within 24 hours.

Search Methods for the Identification of Studies

**Figure 1:**
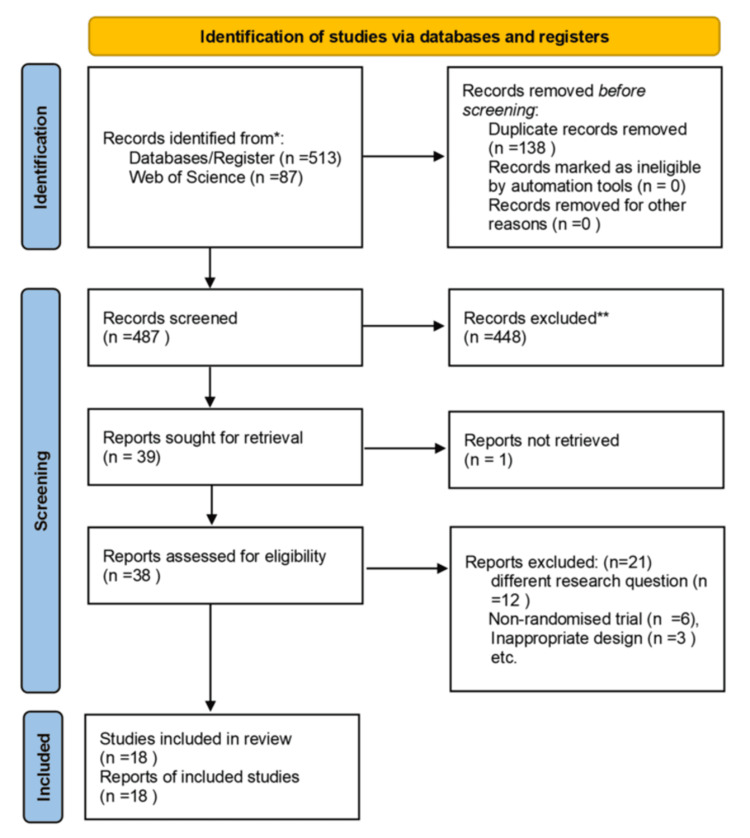
PRISMA flow diagram depicting the selection of studies *Electronic search from Cochrane, PUBMED, EBSCO, and Google scholar. ** Exclusion based on reviewing abstract to check for eligibility criteria PRISMA: Preferred Reporting Items for Systematic Reviews and Meta-Analyses

Electronic search: We started the search for relevant articles on databases such as Cochrane and local electronic libraries. We then extended our search to include MEDLINE, EMBASE, and CINAHL. Additionally, we manually searched other platforms like BMC, the European Journal, and the American Journal for any relevant studies. After screening the titles and abstracts, we searched for protocols and full articles. Any articles that were not freely available were accessed through our local Health Service Executive (HSE) library in Ireland. Figure 1 shows the flow diagram for screening and selecting the studies. Table 1 shows the search strategy.

**Table 1:** Search Strategy For Google Scholar, Web of Science, BMC, American journals, and European journals, we used free-text terms like “outpatient vs. inpatient induction of labor”, and “Induction of labor”. For PubMed, Medline, CINHAL (EBSCO), EMBASE, and Cochrane, we used the search terms presented in this table

Set	Searched for
S10	(S1 OR S2) AND (S3 OR S4 OR S5 OR S6) AND (S7 OR S8 OR S9)
S9	TI,AB("outpatient induction of labor with Foley catheter" OR "mechanical outpatient induction of labor" OR "outpatient IOL with an osmotic Hegar's dilator")
S8	EMB.EXACT("Foley catheter") OR CINAHL.EXACT("Foley catheter") OR COCHRANE.EXACT("Foley catheter")
S7	(RCT OR "Randomized Controlled Trial") OR MESH.EXACT("Randomized Controlled Trials as Topic") OR CINHAL.EXACT("Randomized Controlled Trial")
S6	AB,TI(("outpatient induction of labor" OR "outpatient versus inpatient induction of labor" OR "induction of labor" OR "home induction of labor" OR "inpatient induction of labor"))
S5	EMB.EXACT("Labor Induction") OR MESH.EXACT("Labor, Induced") OR CINAHL.EXACT("Labor Induction") OR COCHRANE.EXACT("Labor Induction")
S4	AB,TI((labor OR delivery) n/1 (induc* OR stimula*)) OR MESH.EXACT("Labor, Obstetric")
S3	EMB.EXACT("Outpatients") OR MESH.EXACT("Outpatients") OR CINHAL.EXACT("Outpatients")
S2	AB,TI("outpatient" OR "outpatient versus inpatient") OR "home induction"
S1	AB,TI(("home induction of labor" OR ambula* OR home)) OR Google Scholar("outpatient induction of labor") OR Web of Science("induction of labor")

Three researchers, MM, MB, and FA, conducted a manual electronic search for studies using keywords related to outpatient induction of labor. The keywords included phrases such as "outpatient induction of labor," "mechanical outpatient induction of labor," "outpatient versus inpatient induction of labor," "outpatient induction of labor with Foley catheter," "outpatient IOL with an osmotic Hegar's dilator," "induction of labor," and "home induction of labor." After the studies were gathered, they were screened by reviewing the titles and abstracts to check if they met the previously established inclusion criteria. There were no restrictions on countries or languages when searching for the studies.

Data were extracted from selected studies and entered manually into RevMan web, as previously identified interventions had already been prepared. Study outcomes were reported as summary risk ratios (RR) for dichotomous outcomes and mean difference (MD) for continuous outcomes, with 95% confidence intervals (CI). A random effect model was used if significant heterogeneity was observed (i.e., I^2^ more than 50%). Additionally, SPSS Statistics version 29 (IBM Corp., Armonk, NY) was utilized to aggregate demographic data from all studies, including age and parity.

Risk of bias assessment in included studies was conducted by two independent authors for each selected study. Both authors reviewed the full text of the studies and utilized the ROB2 tool to assess the study's validity. This involved examining all five domains of ROB2, including the randomization process, allocation sequence, assessment of baseline imbalance, performance bias, outcome bias, missing data, and selective reporting. Any disagreements were resolved through discussion and by referencing the Cochrane Handbook. In some cases, a third or fourth author was consulted, and on one occasion, we sought guidance from a professor at the University of Galway, Ireland.

Risk of Bias in Included Studies

**Figure 2:**
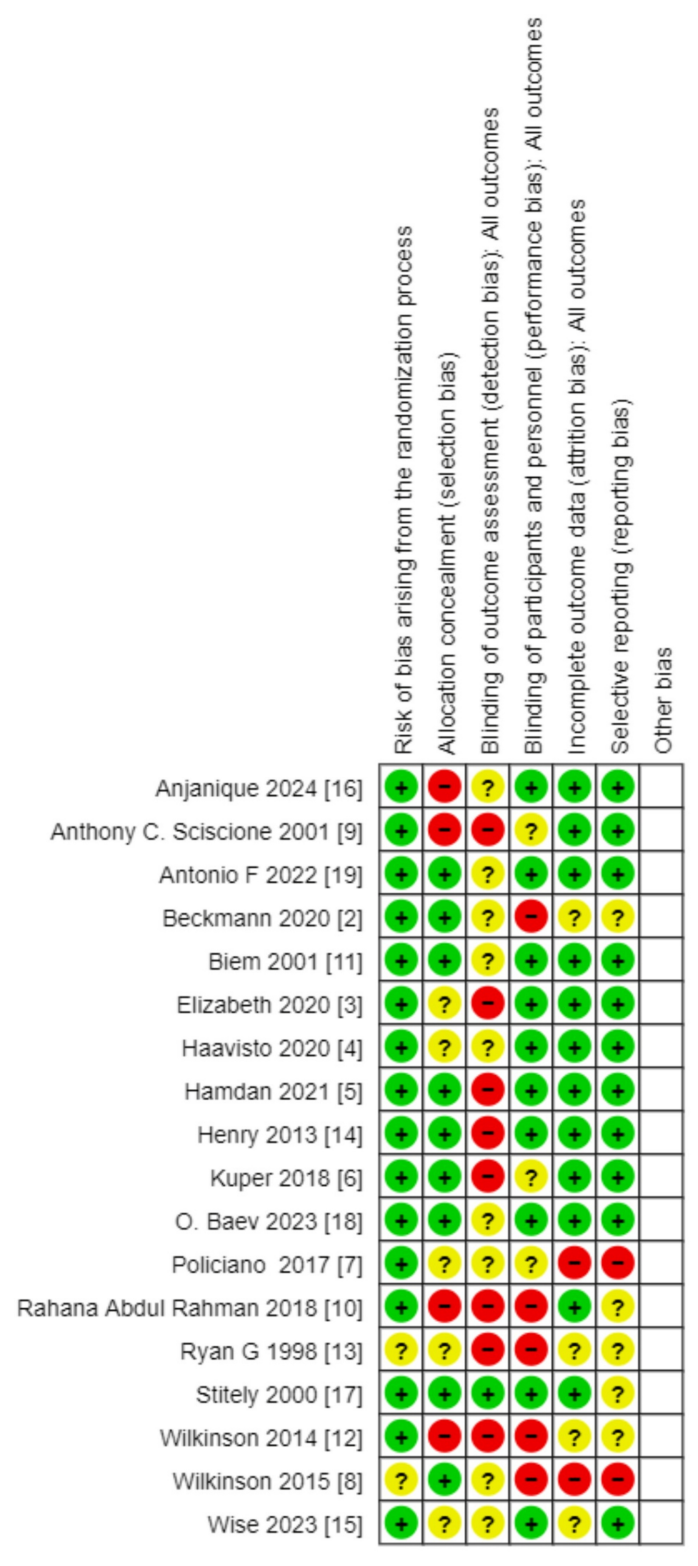
Risk of bias for included studies

**Figure 3:**
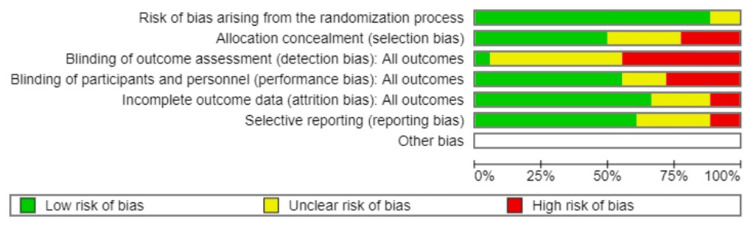
Risk of bias graph for included studies

The risk of bias was assessed according to the ROB2 tool summary of assessment in Figure 2 and Figure 3. (ROB2 tool is included in the RevMan web). The majority of included studies were assessed to be at low risk for bias with the randomization process. Two studies were assessed to have unclear risk: Ryan et al. [13] and Wilkinson et al. [8]. Most of the studies mentioned the process for allocation. The studies with unclear or high risk were Lu et al. [16], Sciscione et al. [9], Ausbeck et al. [3], Haavisto et al. [4], Policiano et al. [7], Rahman et al. [10], Ryan et al. [13], Wilkinson et al. [12], and Wise et al. [15]. The included studies in this analysis were all open-labeled and not blinded. Four studies with unclear risk were Sciscione et al. [9], Beckmann et al. [2], Ryan et al. [13], and Wilkinson et al. [12]. Two studies were judged to be at high risk of attrition bias: Policiano et al. [7] and Wilkinson et al. [8]. Most of the studies were assessed to be at low risk for selective reporting. Studies with unclear or high risk of bias included Beckmann et al. [2], Policiano et al. [7], Rahman et al. [10], Ryan et al. [13], Stitely et al. [17], and Wilkinson et al. [12].

Excluded Studies

Studies by Bhide et al. [20], Kruit et al. [21], Hallén et al, [22], PonMalar et al. [23], Hong et al. [24], O'Brien et al. [25], Merollini and Beckmann [26]; Jozwiak et al. [27]; Ten Eikelder et al. [28], Kandahari et al. [29], Kadu and Shiragur [30], Adelson et al. [31], Salvador et al. [32], McGee et al. [33], Gaffaney et al. [34], Elliot et al. [35], Incerpi et al. [36], McKenna et al. [37], Seasely et al. [38], Bollapragada et al. [39], Kipikasa et al. [40], were excluded due to issues related to methodology, objectives, or the outcomes reported.

Statistical heterogeneity was assessed using I^2^ and interpreted according to Cochrane guidelines. We used a cut-off of 50% to categorize results with high heterogeneity. With significant heterogeneity, we used the random effect model; additionally, we investigated sensitivity within forest plots. Sensitivity analysis was performed with the exclusion of studies with a high risk of bias where possible. The GRADE approach was used to assess the certainty of the evidence.

Results

Table 2 shows the characteristics of the included studies.

**Table 2:** Characteristics of included studies CS: cesarean section; CTG: cardiotocograph; GBS: group B Streptococcus; HBV: hepatitis B virus; HIV: human immunodeficiency virus; IOL: induction of labor; NICU: neonatal intensive care unit; PGE2: prostaglandin E_2_; PPH: postpartum hemorrhage; RCT: randomized controlled trial; SGA: small for gestational age

Study	Methods	Participants	Interventions	Outcomes	Inclusion criteria	Exclusion criteria
Lu et al., 2024 [16]	Open-label parallel RCT	Pregnant women low risk; multipara and primipara; with a Bishop score <6	Outpatient IOL by hygroscopic dilator and Foley catheter	Time from admission to delivery, maternal and neonatal outcomes	Age 18-40, gestation >37 weeks, Bishops score less than 6, cervical dilatation <2 cm, and reactive CTG	Not able to consent in English, high risk/placental insufficiency, SGA, preeclampsia GBS carrier, HIV/HBV, Bishop score more than 6, and allergy to induction methods, e.g., Foley catheter
Sciscione et al., 2001 [9]	Open-label RCT	Low-risk pregnant women with a Bishop score <6	Outpatient versus inpatient IOL with Foley catheter	Change in Bishop score, CS rate, and NICU admission	More than 37 weeks gestation, single cephalic, Bishop score of less than 5	Placenta previa, low-lying placenta, undiagnosed vaginal bleeding, preeclampsia, fetal anomaly, growth restriction, rupture of membrane, excessive distance (more than 30 minutes), and unreliable transportation
Sciscione et al., 2022 [19]	Open-label parallel RCT	Women low risk at term planned for IOL. 339 women were enrolled; 171 received inpatient IOL by Hegar's dilator and 167 by outpatient Hegar's	Outpatient compared with inpatient preinduction cervical ripening using a synthetic osmotic dilator	The primary outcome was the rate of hospital stays longer than 48. Secondary outcomes were labor, delivery, and neonatal outcomes	More than 18 yrs of age, gestational age more than 37 weeks, cephalic presentation, cervix less than 3 cm dilated	Active labor, genital herpes, chorioamnionitis, previous uterine surgery, previous cesarean delivery, non-reassuring CTG, contraindication to vaginal delivery, and pregnancy-related complications (high-risk populations)
Beckmann et al., 2020 [2]	Open-label parallel RCT	Women with uncomplicated term singleton pregnancies; 695 women enrolled, 215 randomized to an outpatient IOL by Foley catheter, and 233 to inpatient IOL by dinoprostone gel	Balloon-outpatient and PG-inpatient	The primary outcome measure was a composite measure of neonatal outcomes. Healthcare experience was a secondary outcome measure	Singleton, cephalic presentation, gestational age more than 37 weeks, low-risk pregnancy	Major congenital anomalies, SGA, previous cesarean section, Bishop score more than 7, high head (4/5), and residing more than 60 minutes from the hospital
Biem et al., 2001 [11]	Open-label parallel RCT	Low-risk pregnant women with singleton babies and Bishop score <6. The total number of participants was 300	Outpatient and inpatient PG insert	The primary outcomes were effectiveness and satisfaction. Other outcomes of interest were duration of hospital stay; maternal and neonatal safety including uterine hyperstimulation, abruptio placentae, maternal adverse events, and neonatal intensive care unit admission	Women with single baby, more than 37 weeks, normal CTG, parity less than 5, and Bishop score less than 6	Ruptured membrane, high-risk pregnancies, and fetal congenital anomalies
Elizabeth et al., 2020 [3]	Open-label parallel RCT	Women at term low risk, first pregnancy, planned for IOL; the total number of women was 126 randomized to inpatient and outpatient IOL by Foley catheter; both groups were similar in prognostic factors	Outpatient and inpatient IOL by Foley catheter	Chorioamnionitis, endometritis, cesarean delivery, post-partum hemorrhage, delivery within 24 hours of hospital admission, total hospital length of stay, and hospital readmission within 30 days of oxytocin infusion and meconium-stained amniotic fluid, time from rupture of membranes to delivery, intrapartum temperature	Nulliparous, more than 37 weeks, more than 18 years of age, singleton cephalic fetus, reliable transportation, and Bishop score less than 6	High-risk pregnancies with prespecified medical or obstetric complication
Haavisto et al., 2020 [4]	Open-label parallel RCT	Low-risk pregnant women at term with Bishop score <6; the total number of participants was 113 randomized to inpatient/outpatient IOL by Foley catheter	Outpatient/inpatient IOL by Foley catheter	Maternal satisfaction and obstetrics outcome	Singleton, cephalic presentation fetuses, more than 37 weeks, uncomplicated pregnancy, intact membranes, a Bishop score <6, normal CTG, short distance to the hospital (at most, half an hour drive), and sufficient knowledge of the Finnish language	A medical condition of pregnancy and SGA
Hamdan et al., 2021 [5]	Open-label parallel RCT	Term low-risk population with Bishop score <6; the total number of women enrolled was 163 randomized to outpatient IOL by Foley catheter (n=82) and inpatient IOL (n=81)	Outpatient IOL/inpatient IOL with Foley catheter	Delivery during “working hours” 08:00–18:00 h and maternal satisfaction	Multiparous, more than 18 years, gestational age more than 37 weeks, singleton, cephalic fetus, intact membrane, normal CTG, uncomplicated pregnancy close to hospital, and Bishop score less than 5	Fetal anomalies, allergy to latex, previous uterine surgery
Henry et al., 2013 [14]	Open-label parallel RCT	Low-risk women with unfavorable cervix planned for IOL, including 91 nulliparas and 10 multiparas	A total number of 101 patients, were randomized to outpatient induction with Foley catheter (n=50) and inpatient induction with prostaglandin gel (n=51)	Mode of birth, induction to delivery interval, vaginal delivery within 24 hours of commencement of cervical ripening, percentage of women requiring oxytocin, and total inpatient stay	More than 18 years, gestational age more than 37 weeks, normal CTG, Bishop less than 6	Unsuitable for outpatient IOL, not suitable for hormonal induction, Bishop score more than 7, and multiple pregnancies
Kuper et al., 2018 [6]	Open-label RCT	Parous women at 39 weeks of gestation or greater with a cervix 3 cm or less dilated; if 2–3 cm dilated, less than 80% effaced and reassuring fetal heart rate monitoring	Women were randomized in the ambulatory setting to either an outpatient transcervical catheter (with immediate placement) or inpatient transcervical catheter placement and concomitant oxytocin infusion in the labor ward	The primary outcome was the total duration of time from admission to the labor ward until delivery. Secondary analysis included total hospital duration. Other outcomes included rates of acetaminophen use, admissions before the scheduled admission time, and spontaneous rupture of amniotic membranes	Low-risk parous, 18 years old with a singleton fetus in vertex presentation, gestational age more than 39 weeks	High-risk pregnancies, Bishop score more than 7, and abnormal CTG
Baev et al., 2023 [18]	Open-label parallel RCT	Women at term low risk, with singleton babies and Bishop score <6; 322 women were enrolled, 160 received inpatient IOL with mifepristone and 162 received outpatient IOL with mifepristone	Mifepristone for outpatient compared to inpatient IOL by same	The primary outcomes included Bishop score improvement, additional use of PGE2, and/or mechanical methods for cervical ripening; additional use of oxytocin; the interval from cervical ripening start to labor onset; labor duration; operative delivery rate; and the total hospital length of stay	Age more than 18 years, singleton fetus, cephalic, gestational age more than 39, intact membranes, no contraindication for vaginal deliveries, and Bishop score less than 6	Noncephalic presentation, high-risk pregnancies, ruptured membrane, previous uterine surgery, and concerns about fetal wellbeing
Policiano et al., 2017 [7]	Open-label parallel RCT	Low-risk women at term for planned IOL; the total number of women was 130; 65 were randomized to outpatient IOL with Foley catheter and 65 were randomized to inpatient IOL by Foley catheter	Outpatient versus inpatient cervix priming with Foley catheter	The primary outcome was the change in the Bishop score	Bishop score less than 6, single fetus, cephalic presentation, gestational age more than 41 weeks	Non-cephalic, contraindication to vaginal delivery, and high-risk pregnancies
Rahman et al., 2018 [10]	Open-label RCT	Low-risk pregnant women with low Bishop scores, and more than 37 weeks	Outpatient and inpatient IOL by Foley catheter	The primary outcomes assessed were cesarean section rate and neonatal sepsis. Secondary outcomes assessed were time from induction to delivery, delivery within 24 hours, duration of inpatient stay, total blood loss, intrapartum pyrexia, primary postpartum hemorrhage, neonatal birth weight, arterial cord pH, meconium aspiration syndrome and admission to the NICU	Single fetus, gestational age beyond 37 weeks, cephalic presentation, intact membranes, Bishop score less than 6, age more than 18 years, lives within 10 km from the hospital	Intrauterine death, intrauterine growth restriction, estimated fetal weight >4000 grams, fetal anomalies, abnormal preinduction CTG, non-vertex presentation, unstable lie, multiple pregnancies, sepsis, hypertension, allergic to latex, had a uterine scar, history of antepartum hemorrhage, parity more than six, suspected cephalo-pelvic disproportion and placenta previa
Ryan et al., 1998 [13]	Open-label RCT	Pregnant women at term	Outpatient PGE2 versus inpatient PGE2	Labor, delivery, and neonatal outcomes	NA	NA
Stitely et al., 2000 [17]	Double-blind RCT	Uncomplicated singleton, vertex pregnancies at 41 weeks gestation or later with a Bishop score of 4 or less; 60 patients were enrolled, 27 were randomized to outpatient induction by 25 ug vaginal misoprostol, and 33 to inpatient placebo	Misoprostol intravaginal cervical ripening compared to inpatient placebo	The primary outcome measure was the number of inpatient inductions needed by study day 3	All gravid patients, gestational age more than 41 weeks, and Bishop score less than 4	Multiple pregnancies, non-cephalic, pregnancies with morbidities and medical conditions
Wilkinson et al., 2014 [12]	Open-label parallel RCT	Women at term low risk planned for IOL; the total number of women enrolled and randomized was 827. 411 women were randomized to outpatient IOL by PGE2 gel (168 completed the intervention) and 416 (210 completed the intervention) to inpatient PGE2 gel	Inpatient/outpatient IOL by PGE2	Oxytocin use, maternal and fetal outcomes, and whether planned outpatient management was achievable	Uncomplicated pregnancies, more than 18 years, more than 41 weeks, cephalic, singleton pregnancies, no previous cesarean section	High-risk pregnancies and SGA
Wilkinson et al., 2015 [8]	Open-label parallel RCT	Women at term for IOL; 48 women were randomized, 15 to inpatient IOL by Foley catheter and 33 to outpatient IOL by Foley catheter	IOL by Foley catheter inpatient/outpatient	Obstetrics outcome and satisfaction	Term (37–42 weeks), healthy pregnancy; intact membranes and Bishop score of <7; singleton, cephalic presentation and appropriately grown	Contraindications for vaginal delivery
Wise et al., 2023 [15]	Open-label parallel RCT	Pregnant women with singleton pregnancy >37 weeks, low risk, nullipara and multipara with low Bishop score. 1087 women were enrolled, 548 received IOL inpatient by PGE2 either gel or insert, and 539 received outpatient induction by Foley catheter	Outpatient induction of labor with a Foley catheter and inpatient induction with prostaglandin	Primary outcome: CS rate; secondary outcome: duration of cervical ripening, vaginal delivery within 24 hours, use of oxytocin, use of epidural, induction to ARM, failed induction, PPH, and general neonatal outcome	All gravida patients, more than 37 weeks, Bishop score <7, and fair English	Previous cesarean section, contraindications for vaginal delivery, and fetal compromise

Result of Main Analysis: Outpatient Versus Inpatient Induction of Labor (All Methods Combined)

A total of 18 randomized control trials, with 4886 patients, compared outpatient to inpatient induction using all available methods of induction, either mechanical or hormonal. The mean age for the outpatient group was 29.3 years and that for the inpatient group was 29.2 years.

**Figure 4:**
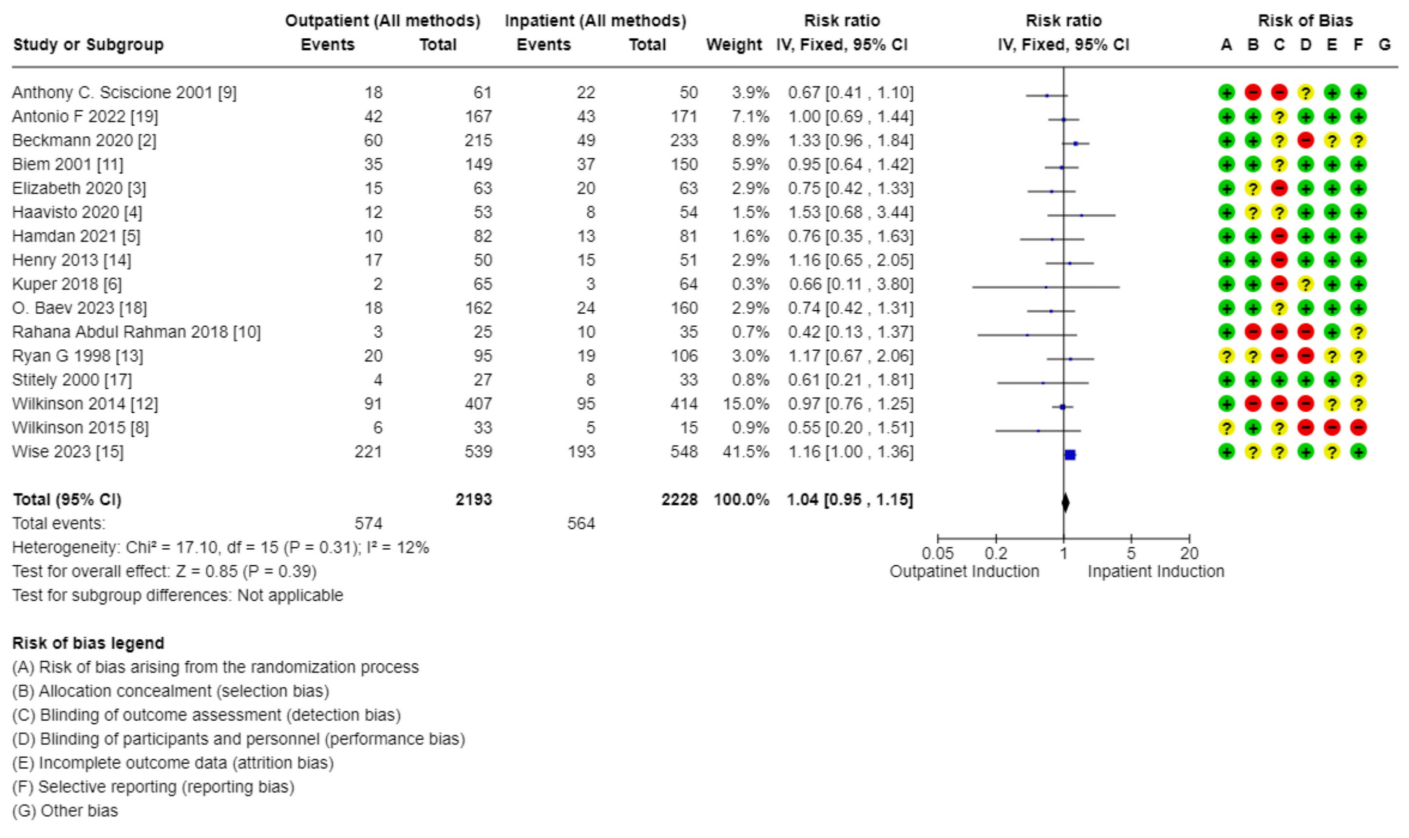
Forest plot for cesarean section (outpatient vs. inpatient induction Of labor)

**Figure 5:**
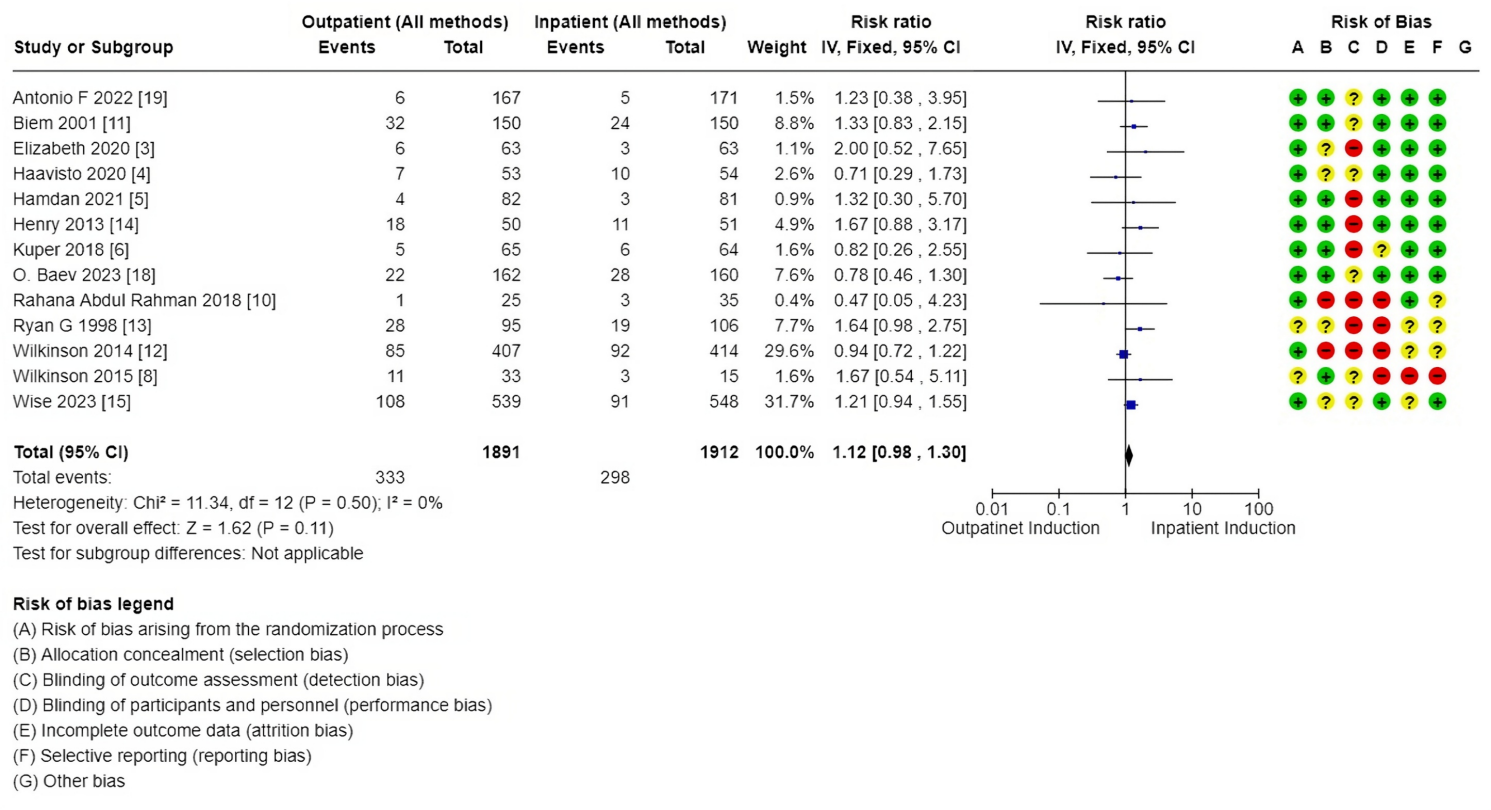
Forest plot for operative vaginal delivery [outpatient vs. inpatient induction Of labor, main analysis (all methods)]

**Figure 6:**
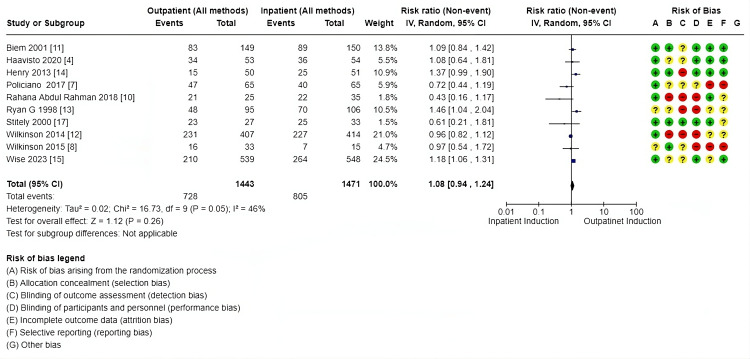
Forest plot for vaginal delivery rate (outpatient vs. inpatient induction of labor, main analysis)

Primary outcomes: There was no difference in cesarean section rate between outpatient versus inpatient induction (4421 patients, 16 RCTs, RR: 1.01, 95% CI: 0.90-1.14; moderate certainty of evidence) (Figure 4). Also, no difference in operative vaginal delivery (3803 patients, 13 RCTs, RR: 1.12, 95% CI: 0.98-1.30; moderate certainty of evidence) (Figure 5), and no difference in vaginal delivery rate (2914 patients, 10 RCTs, RR: 1.08, 95% CI: 0.94-1.24; low certainty of evidence, as shown in the GRADE approach summary in Tables 3-4) was observed as well. Demonstration of the analysis is shown in the forest plots in Figures 4-6.

**Table 3:** Summary of findings for outpatient vs. inpatient induction of labor (all methods) ^a^As most of the included studies were open-label, there were concerns about the allocation concealment and the blinding of the outcome assessors ^b^There were concerns about incomplete data and selective reporting ^c^There was significant heterogeneity and a large difference in the actual assessment of the effect, with confidence intervals not overlapping in most of the studies ^d^Funnel plots suggested publication bias ^e^A wide confidence interval includes the possibility of no effect or appreciable harm or benefit ^f^Febrile morbidities in some studies were not directly related to the intrapartum period, but a few were associated with the immediate postpartum period ^g^Optimum size information not met Assessment of indirectness was done directly with the GRADE table where most of the included studies were deemed to answer the research question within the relevant population. Imprecision was assessed using the confidence interval approach (wide CI if 0.70 and 1.30 included), optimal information size, and the number of events. Inconsistency was assessed for any individual outcome by assessing confidence interval variations and heterogeneity CI: confidence interval; MD: mean difference; RCT: randomized control trial; RR: risk ratio; SCBU: special care baby unit

Certainty assessment							Summary of findings				
Participant (studies) follow-up	Risk of bias	Inconsistency	Indirectness	Imprecision	Publication bias	Overall certainty of evidence	Study event rates (%)		Relative effect (95% CI)	Anticipated absolute effects	
							With inpatient (control)	With outpatient		Risk with inpatient (control)	Risk difference with outpatient
Cesarean section rate											
4421 (16 RCTs)	Serious^a^	Not serious	Not serious	Not serious	None	⨁⨁⨁◯ Moderate	564/2228 (25.3%)	574/2193 (26.2%)	RR: 1.04 (0.95 to 1.15)	564/2228 (25.3%)	10 more per 1000 (from 13 fewer to 38 more)
Operative vaginal delivery rate											
3803 (13 RCTs)	Serious^a^	Not serious	Not serious	Not serious	None	⨁⨁⨁◯ Moderate	298/1912 (15.6%)	333/1891 (17.6%)	RR: 1.12 (0.98 to 1.30)	298/1912 (15.6%)	19 more per 1000 (from 3 fewer to 47 more)
Vaginal delivery rate											
2914 (10 RCTs)	Serious^b^	Serious^c^	Not serious	Not serious	Publication bias strongly suspected^d^	⨁◯◯◯ Very low	805/1471 (54.7%)	728/1443 (50.5%)	RR: 1.08 (0.94 to 1.24)	805/1471 (54.7%)	44 more per 1000 (from 33 fewer to 131 more)
Additional usage of another induction agent											
2195 (7 RCTs)	Serious	Serious^c^	Not serious	Not serious	Publication bias strongly suspected^d^	⨁◯◯◯ Very low	182/1094 (16.6%)	280/1101 (25.4%)	RR: 1.13 (0.69 to 1.86)	182/1094 (16.6%)	22 more per 1000 (from 52 fewer to 143 more)
SCBU admission											
4128 (13 RCTs)	Serious^a^	Not serious	Not serious	Not serious	None	⨁⨁⨁◯ Moderate	136/2076 (6.6%)	131/2052 (6.4%)	RR: 0.99 (0.79 to 1.24)	136/2076 (6.6%)	1 fewer per 1000 (from 14 fewer to 16 more)
Failed Induction											
1491 (6 RCTs)	Very serious^a,b,d^	Serious^c^	Not serious	Serious^e^	Publication bias strongly suspected^d^	⨁◯◯◯ Very low	76/728 (10.4%)	71/763 (9.3%)	RR: 0.81 (0.38 to 1.73)	76/728 (10.4%)	20 fewer per 1000 (from 65 fewer to 76 more)
Febrile Mirbdiity											
904 (6 RCTs)	Very serious^a,b^	Not serious	Serious^f^	Serious^e^	Publication bias strongly suspected^d^	⨁◯◯◯ Very low	40/446 (9.0%)	54/458 (11.8%)	RR: 1.30 (0.89 to 1.90)	40/446 (9.0%)	27 more per 1000 (from 10 fewer to 81 more)
Induction to delivery (hours)											
1211 (8 RCTs)	Serious^a^	Serious^c^	Not serious	Serious	Publication bias strongly suspected^d^	⨁◯◯◯ Very low	592	619	-	592	MD: 5.61 higher (2.5 lower to 13.73 higher)
The interval from induction to labor onset (hours)											
669 (3 RCTs)	Very serious^a,b^	Not serious	Not serious	Serious^g^	Publication bias strongly suspected^d^	⨁◯◯◯ Very low	325	344	-	325	MD: 1.59 lower (1.93 lower to 1.26 lower)
Interval from admission to delivery (hours)											
275 (3 RCTs)	Serious^a^	Not serious	Not serious	Serious^g^	Publication bias strongly suspected^d^	⨁◯◯◯ Very low	129	146	-	129	MD: 6.92 lower (10.39 lower to 3.45 lower)
Oxytocin usage											
2813 (10 RCTs)	Serious^a,b^	Not serious	Not serious	Not serious	Publication bias strongly suspected^d^	⨁⨁◯◯ Low	726/1410 (51.5%)	772/1403 (55.0%)	RR: 0.98 (0.85 to 1.13)	726/1410 (51.5%)	10 fewer per 1000 (from 77 fewer to 67 more)
Postpartum hemorrhage											
2376 (8 RCTs)	Very serious^a,b^	Not serious	Not serious	Not serious	Publication bias strongly suspected^d^	⨁◯◯◯ Very low	259/1187 (21.8%)	278/1189 (23.4%)	RR: 1.07 (0.93 to 1.22)	259/1187 (21.8%)	15 more per 1000 (from 15 fewer to 48 more)
Spontaneous rupture of membranes											
858 (5 RCTs)	Serious^a^	Serious^c^	Not serious	Not serious	Publication bias strongly suspected^d^	⨁◯◯◯ Very low	73/429 (17.0%)	36/429 (8.4%)	RR: 1.04 (0.95 to 1.15)	73/429 (17.0%)	7 more per 1000 (from 9 fewer to 26 more)
Total hospital stay (hours)											
3455 (11 RCTs)	Serious^a^	Serious^c^	Not serious	Not serious	Publication bias strongly suspected^a^	⨁◯◯◯ Very low	1748	1707	-	1748	MD: 5.68 lower (8.69 lower to 2.67 lower)
Uterine hyperstimulation											
2308 (4 RCTs)	Serious^a,b^	Not serious	Not serious	Serious^e^	Publication bias strongly suspected^d^	⨁◯◯◯ Very low	86/1163 (7.4%)	89/1145 (7.8%)	RR: 1.06 (0.80 to 1.40)	86/1163 (7.4%)	4 more per 1000 (from 15 fewer to 30 more)
Vaginal birth within 24 hours											
1673 (4 RCTs)	Very serious^a,b^	Serious^c^	Not serious	Not serious	Publication bias strongly suspected^d^	⨁◯◯◯ Very low	302/844 (35.8%)	176/829 (21.2%)	RR: 0.63 (0.39 to 1.02)	302/844 (35.8%)	132 fewer per 1000 (from 218 fewer to 7 more)

**Table 4:** Summary of the results for outpatient vs. inpatient induction with all methods combined CI: confidence interval; MD: mean difference; RR: risk ratio

Analysis title	No. of studies	No. of participants	Statistical method	Effect size
Cesarean section rate	16	4421	RR (IV, random, 95% CI)	1.01 [0.90, 1.14]
Operative vaginal delivery rate	13	3803	RR (IV, random, 95% CI)	1.12 [0.98, 1.30]
Vaginal delivery rate	10	2914	RR (IV, random, 95% CI)	1.08 [0.94, 1.24]
Additional usage of another induction agent	7	2195	RR (IV, random, 95% CI)	1.13 [0.69, 1.86]
SCBU admission	13	4128	RR (IV, fixed, 95% CI)	0.99 [0.79, 1.24]
Failed induction	6	1491	RR (IV, random, 95% CI)	0.81 [0.38, 1.73]
Febrile morbidity	6	904	RR (IV, random, 95% CI)	1.30 [0.89, 1.90]
Induction to delivery (hours)	8	1211	MD (IV, random, 95% CI)	5.61 [-2.50, 13.73]
The interval from induction to labor onset (hours)	3	669	MD (IV, fixed, 95% CI)	-1.59 [-1.93, -1.26]
Interval from admission to delivery (hours)	3	275	MD (IV, random, 95% CI)	-6.92 [-10.39, -3.45]
Oxytocin usage	10	2813	RR (IV, random, 95% CI)	0.98 [0.85, 1.13]
Postpartum hemorrhage	8	2376	RR (IV, random, 95% CI)	1.07 [0.93, 1.22]
Spontanous rupture of membrane	5	858	RR (IV, random, 95% CI)	1.04 [0.95, 1.15]
Total hospital stay (hours)	11	3455	MD (IV, random, 95% CI)	-5.68 [-8.69, -2.67]
Uterine hyperstimulation	4	2308	RR (IV, random, 95% CI)	1.06 [0.80, 1.40]
Vaginal birth within 24 hours	4	1673	RR (IV, random, 95% CI)	0.63 [0.39, 1.02]

**Figure 7:**
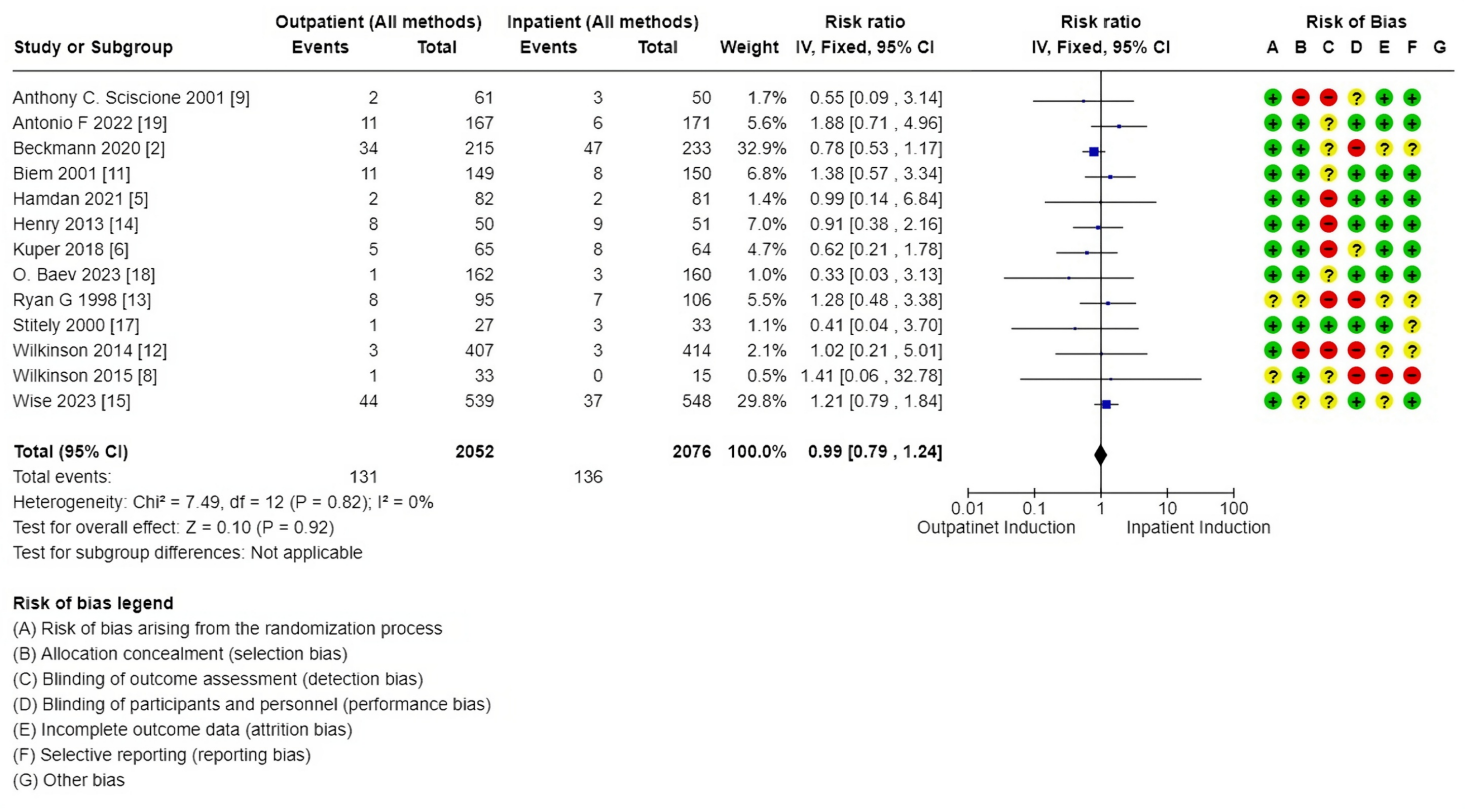
Forest plot for special care baby unit admission (outpatient vs. inpatient induction of labor, main analysis)

**Figure 8:**
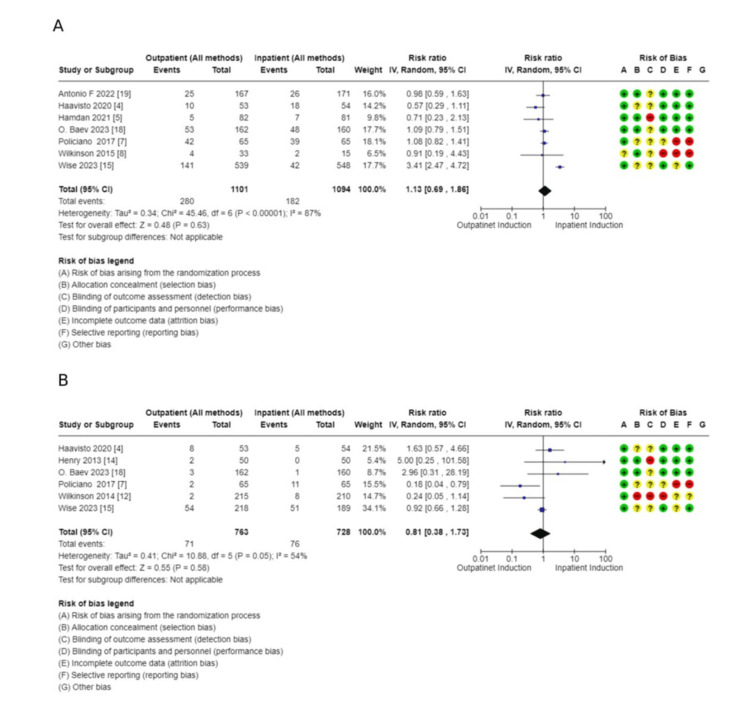
Secondary outcome forest plots - 1 A: additional usage of an induction agent; B: failed induction

**Figure 9:**
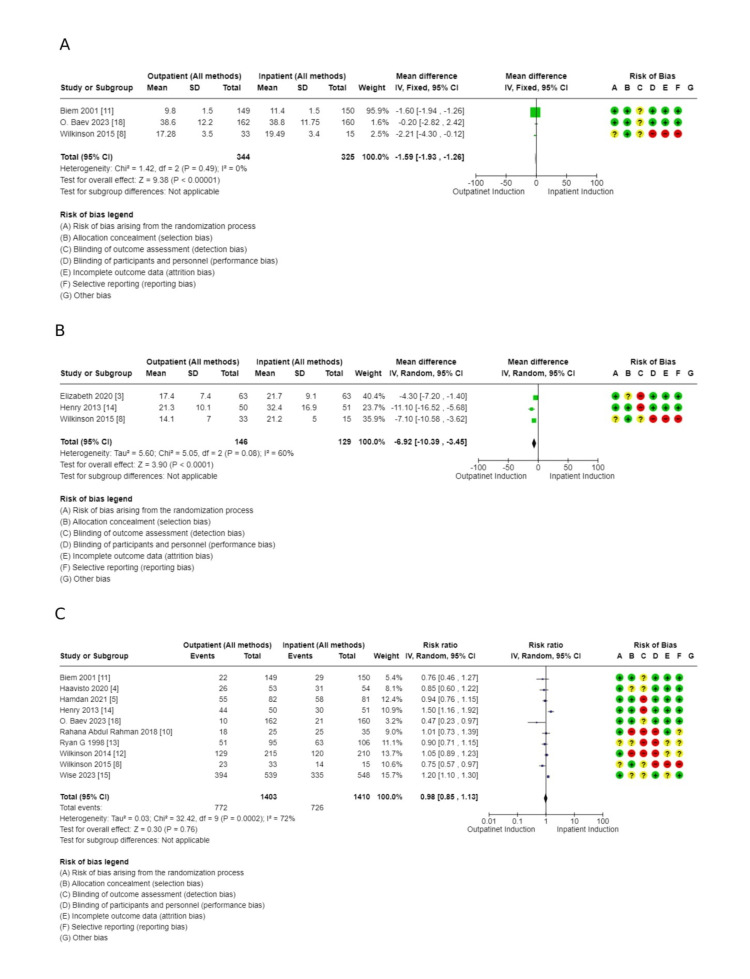
Secondary outcome forest plots - 2 A: the interval from labor induction to labor onset; B: the interval from admission to delivery; C: oxytocin usage

**Figure 10:**
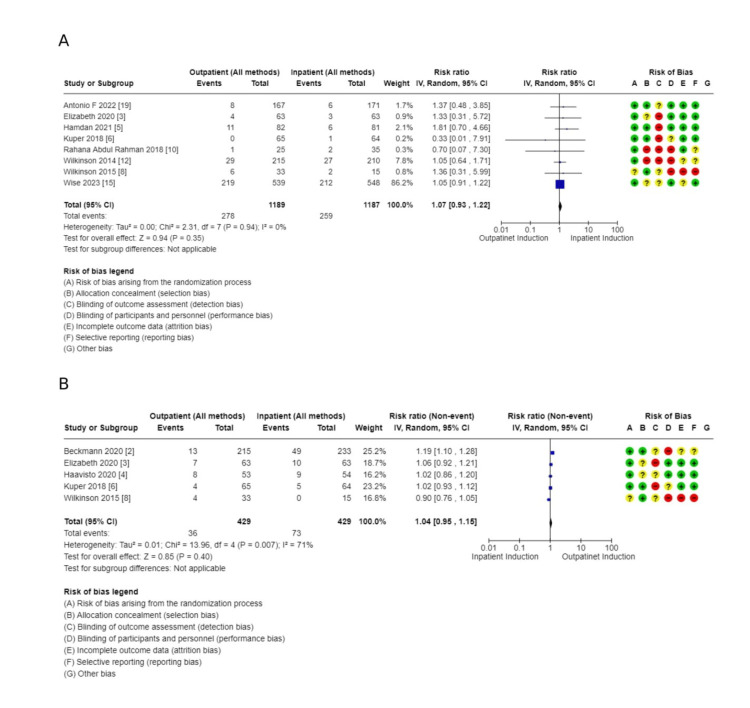
Secondary outcome forest plots - 3 A: postpartum haemorrhage rate; B: spontaneous rupture of membrane

**Figure 11:**
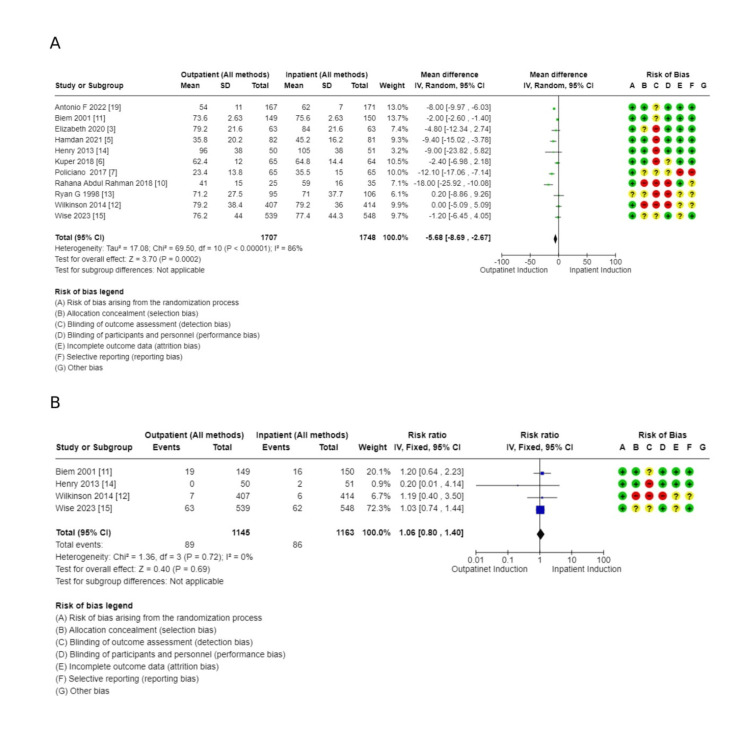
Secondary outcomes to main analysis A: total hospital stay; B: uterine hyperstimulation

Secondary outcomes: These outcomes showed little or no difference, including additional usage of another induction agent (2914, 10 RCTs, RR: 1.08, 95% CI: 09.4-1.2), failed induction (1491, 6 RCTs, RR: 0.81, 95% CI: 038-173), oxytocin usage (2813, 10 RCTs, RR: 0.98, 95% CI: 0.85-1.13), spontaneous rupture of membrane (858, 5 RCTs, RR: 1.04, 95% CI: 0.95-1.15), febrile morbidity (904, 6 RCTs, RR: 1.30, 95% CI: 0.89-1.90), the induction to delivery interval in hours (5.61 higher 2.5-13.73), vaginal delivery within 24 hours (1673, 4 RCTs, RR: 0.63, 95% CI: 0.39-1.02, and SCBU admission (4128, 13 RCTs, RR: 0.99, 95% CI: 0.79-1.24) (Figures 7-11).

Outcomes related to outpatient induction: interval from induction to labor onset (hours): (1.59 lower, 1.93-1.26 lower); interval from admission to delivery (hours): (6.92 lower, 10.39-3.45 lower), and total hospital stay: (MD: 5.68 hours lower, 8.69-2.67 hours). Full details are shown in Tables 3-4. Forest plots for secondary outcomes are demonstrated in Figures 7-11.

Reporting Bias Assessment

**Figure 12:**
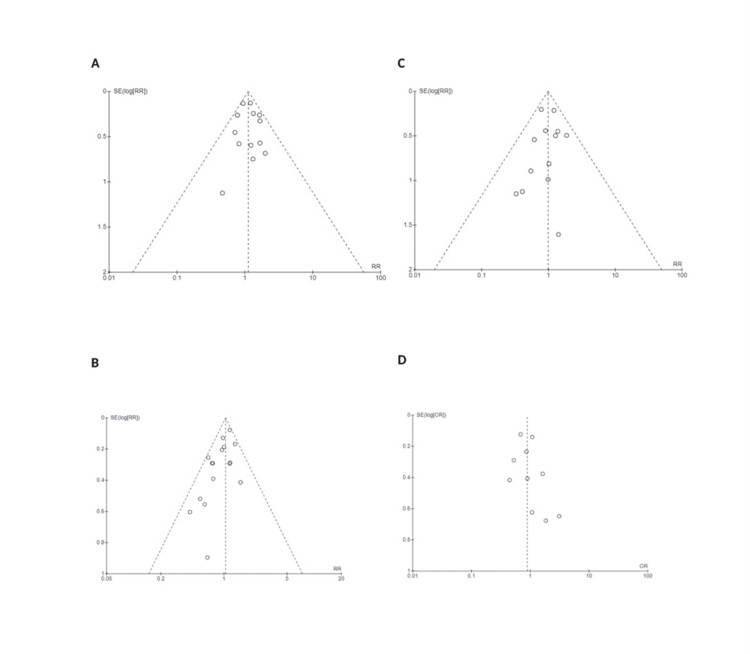
Funnel plot for primary outcomes Funnel plot for publication bias: A: Funnel plot for cesarean section rate, showing symmetrical pyramidal shape, which indicate no publication bias. The Y-axis stands for standard deviation and the X-axis for risk ratio; the blue line indicates 95% CI B: Operative vaginal delivery funnel plots display a symmetrical pyramidal shape, indicating no publication bias. The Y-axis represents standard deviation, and the X-axis represents RR (risk ratio). The blue line represents 95% CI C: Special care baby admission funnel plots display a symmetrical pyramidal shape, indicating no publication bias. The Y-axis represents the standard deviation and the X-axis represents the risk ratio. The blue line represents the 95% CI D: Vaginal delivery rate funnel plot displays an asymmetrical shape, indicating publication bias. The Y-axis represents the standard deviation and the X-axis represents the risk ratio. The blue line represents the 95% CI

We investigated reporting bias using funnel plots for the primary and secondary outcomes. No publication bias was observed in the funnel plot (symmetrical) for the main outcome, as shown in Figure 12. Other secondary outcomes showed asymmetry, which indicates the presence of reporting bias, and those were subsequently downgraded as evidence.

Subgroup Analysis

**Figure 13:**
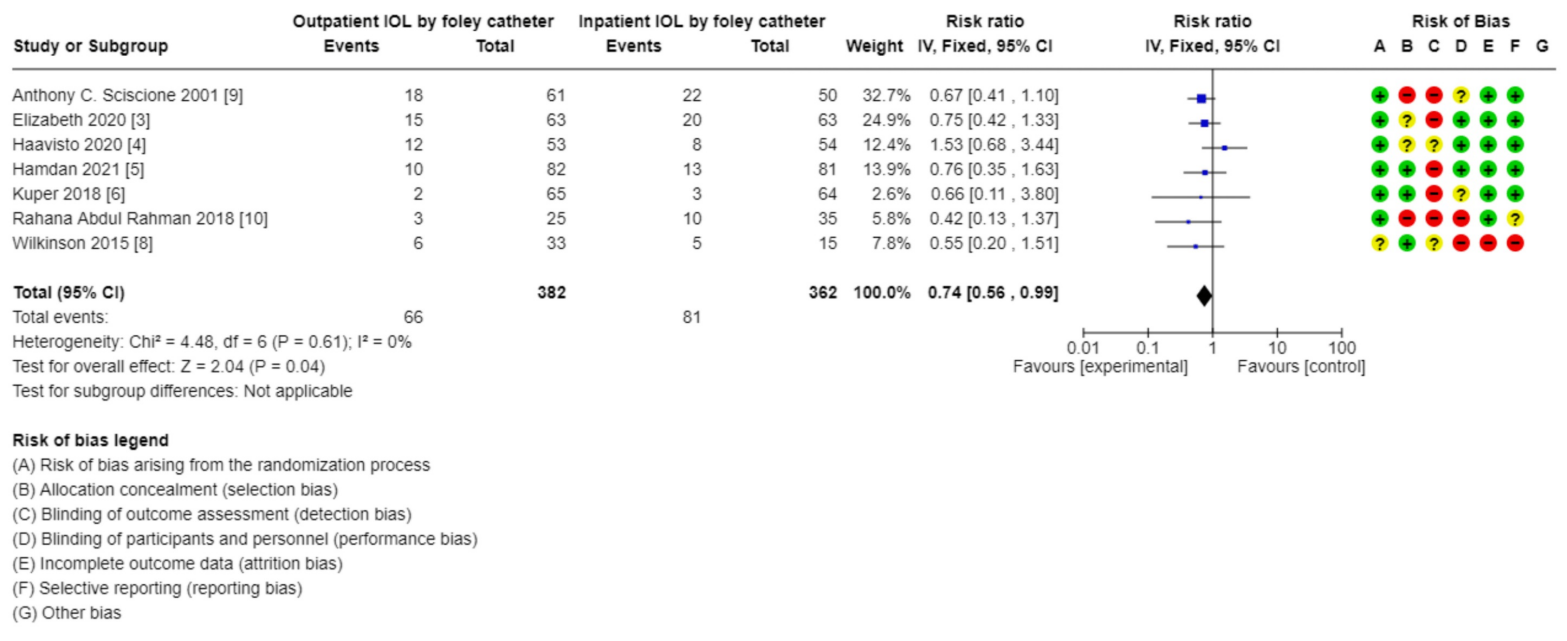
Subgroup analysis: cesarean section rate - outpatient vs. inpatient induction with Foley catheter

**Figure 14:**
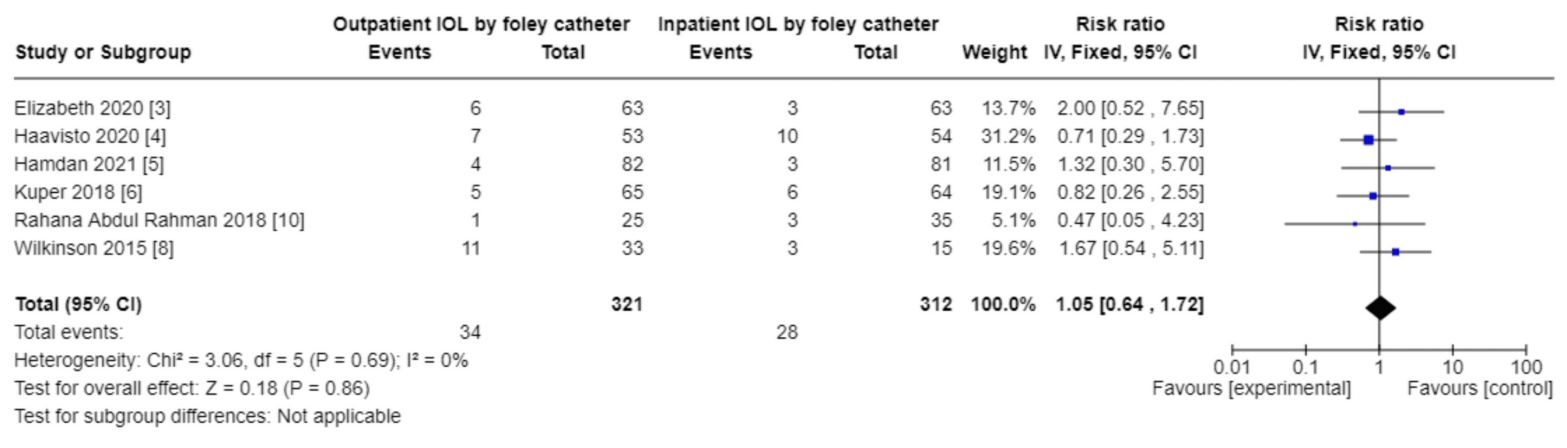
Subgroup analysis: operative vaginal delivery - outpatient vs. inpatient induction with Foley catheter

**Figure 15:**
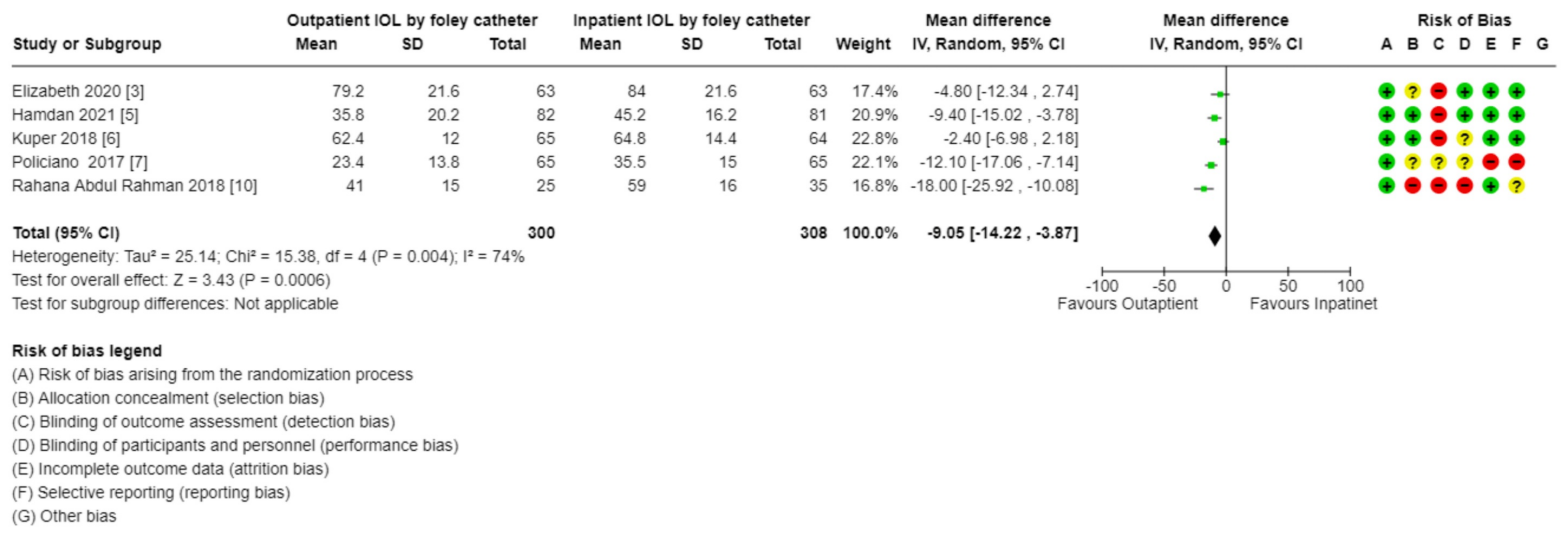
Subgroup analysis: total hospital stay - outpatient vs. inpatient induction with Foley catheter

Comparison of outpatient induction with Foley catheter versus inpatient induction with Foley catheter: There was a reduction in Cesarean section rate in outpatient induction with Foley catheter compared to inpatient induction (744, 7 RCTs, RR: 0.74, 95% CI: 056-0.99), and Induction to delivery interval (hours): (MD: 2.49 lower, 4.48-0.051, low certainty of evidence as shown in Table 5 and Figures 13-15), in addition to a reduction in total hospital stay (608, 5 RCTs, MD: 9.05 lower, 14.22 lower to 3.87, with low certainty of evidence). No difference was found between outpatient versus inpatient induction with Foley catheter in operative vaginal delivery (633, 6 RCTs, RR: 1.5, 95% CI: 0.64-172), postpartum hemorrhage (526, 5 RCTs, RR: 1.39, 95% CI: 0.72-2.68), additional use of another induction agent (448, 4RCTs, RR: 0.92, 95% CI: 0.68-1.25), and spontaneous rupture of membrane (410, 4 RCTs, RR: 0.85, 95% CI: 0.4-1.47). The results are summarized in Table 5.

**Table 5:** Summary of findings for outpatient vs. inpatient induction using Foley catheter ^a^As most of the included studies were open-label, there were concerns about allocation concealment and the blinding of the outcome assessors ^b^Wide confidence interval includes the possibility of no effect or appreciable harm or benefit ^c^There were concerns about incomplete data and selective reporting ^d^Funnel plots suggested publication bias ^e^There was significant heterogeneity and a large difference in the actual assessment of the effect, with confidence intervals not overlapping in most of the studies CI: confidence interval; IOL: induction of labor; MD: mean difference; RCT: randomized control trial; RR: risk ratio

Certainty assessment							Summary of findings				
Participant (studies) follow-up	Risk of bias	Inconsistency	Indirectness	Imprecision	Publication bias	Overall certainty of evidence	Study event rates (%)		Relative effect (95% CI)	Anticipated absolute effects	
							With inpatient IOL by Foley catheter	With outpatient		Risks with inpatient IOL by Foley catheter	Risk difference with outpatient
Cesarean section											
744 (7 RCTs)	Serious^a^	Not serious	Not serious	Serious^b^	None	⨁⨁◯◯ Low	81/362 (22.4%)	66/382 (17.3%)	RR: 0.74 (0.56 to 0.99)	81/362 (22.4%)	58 fewer per 1000 (from 98 fewer to 2 fewer)
Postpartum hemorrhage											
526 (5 RCTs)	Serious^a,c^	Not serious	Not serious	Very serious^b^	Publication bias strongly suspected^d^	⨁◯◯◯ Very low	14/258 (5.4%)	22/268 (8.2%)	RR: 1.39 (0.72 to 2.68)	14/258 (5.4%)	21 more per 1000 (from 15 fewer to 91 more)
Induction to delivery time (hours)											
401 (4 RCTs)	Serious^a,c^	Not serious	Not serious	Not serious	Publication bias strongly suspected^d^	⨁⨁◯◯ Low	196	205	-	196	MD: 2.23 lower (3.83 lower to 0.63 lower)
Operative vaginal delivery											
633 (6 RCTs)	Very serious^a,c^	Not serious	Not serious	Serious^b^	Publication bias strongly suspected^d^	⨁◯◯◯ Very low	28/312 (9.0%)	34/321 (10.6%)	RR: 1.05 (0.64 to 1.72)	28/312 (9.0%)	4 more per 1000 (from 32 fewer to 65 more)
Total hospital stay											
608 (5 RCTs)	Very serious^a,c^	Serious^e^	Not serious	Not serious	Publication bias strongly suspected^d^	⨁◯◯◯ Very low	308	300	-	308	MD: 9.05 lower (14.22 lower to 3.87 lower)
Spontaneous rupture of membrane											
410 (4 RCTs)	Very serious^a,c^	Not serious	Not serious	Serious^b^	Publication bias strongly suspected^d^	⨁◯◯◯ Very low	24/196 (12.2%)	23/214 (10.7%)	RR: 0.85 (0.49 to 1.47)	24/196 (12.2%)	18 fewer per 1000 (from 62 fewer to 58 more)
Additional use of another inducing agent											
448 (4 RCTs)	Very serious^a,c^	Not serious	Not serious	Not serious	Publication bias strongly suspected all plausible residual confounding would reduce the demonstrated effect^d^	⨁⨁◯◯ Low	66/215 (30.7%)	61/233 (26.2%)	RR: 0.97 (0.76 to 1.23)	66/215 (30.7%)	9 fewer per 1000 (from 74 fewer to 71 more)

**Figure 16:**
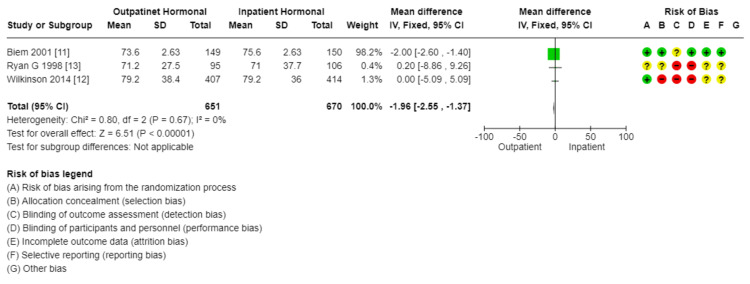
Subgroup analysis: total hospital stay (hours) - outpatient vs. inpatient induction with hormonal methods

Comparison of outpatient versus inpatient induction with prostaglandins (gel or insert): There was little reduction in total hospital stay in outpatient compared to inpatient induction with prostaglandin (MD: 1.96 lower, 2.55 lower to 1.37 lower) (Figure 16). Also, there was no difference between outpatient and inpatient induction with prostaglandin in cesarean section rate (1321, 3 RCTs, RR: 0.99, 95% CI: 0.81 to 1.21), operative vaginal delivery (1322, 3 RCTs, RR: 1.20, 95% CI: 0.85 to 1.70), vaginal delivery rate (1321, 3 RCTs, RR: 0.97, 95% CI: 0.88 to 1.06), uterine hyperstimulation (1120, 2 RCTs, RR: 1.19, 95% CI: 0.69 to 2.05), and SCBU admission (1321, 3 RCTs, RR: 1.28, 95% CI: 0.70 to 2.35). The results are summarized in Table 6.

**Table 6:** Subgroup analysis: outpatient vs. inpatient induction with hormonal methods ^a^As most of the included studies were open-label, there were concerns about allocation concealment and the blinding of the outcome assessors ^b^There were concerns about incomplete data and selective reporting ^c^Funnel plots suggested publication bias ^d^There was significant heterogeneity and a large difference in the actual assessment of the effect, with confidence intervals not overlapping in most of the studies ^e^A wide confidence interval includes the possibility of no effect or appreciable harm or benefit ^f^Special care baby unit (SCBU): if not explicitly mentioned, we considered other neonatal outcomes such as low PH or APGARS, which may indicate the need for admission to the neonatal unit CI: confidence interval; IOL: induction of labor; MD: mean difference; RCT: randomized control trial; RR: risk ratio

Certainty assessment							Summary of findings				
Participant (studies) follow-up	Risk of bias	Inconsistency	Indirectness	Imprecision	Publication bias	Overall certainty of evidence	Study event rates (%)		Relative effect (95% CI)	Anticipated absolute effects	
							With inpatient IOL	With hormonal outpatient		Risks with inpatient IOL	Risk difference with hormonal outpatient
Cesarean section rate											
1643 (4 RCTs)	Very serious^a,b^	Not serious	Not serious	Not serious	Publication bias is strongly suspected; all plausible residual confounding would reduce the demonstrated effect^c^	⨁⨁◯◯ Low	175/830 (21.1%)	164/813 (20.2%)	RR: 0.96 (0.80 to 1.16)	175/830 (21.1%)	8 fewer per 1000 (from 42 fewer to 34 more)
Operative vaginal delivery											
1644 (4 RCTs)	Very serious^a,b^	Serious^d^	Not serious	Not serious	Publication bias strongly suspected^c^	⨁◯◯◯ Very low	163/830 (19.6%)	167/814 (20.5%)	RR: 1.05 (0.86 to 1.27)	163/830 (19.6%)	10 more per 1000 (from 27 fewer to 53 more)
Vaginal delivery											
1321 (3 RCTs)	Very serious^a,b^	Not serious	Not serious	Not serious	Publication bias strongly suspected^c^	⨁◯◯◯ Very low	386/670 (57.6%)	362/651 (55.6%)	RR: 0.93 (0.79 to 1.10)	386/670 (57.6%)	40 fewer per 1000 (from 121 fewer to 58 more)
Uterine hyperstimulation rate											
1120 (2 RCTs)	Very serious^a,b^	Not serious	Not serious	Very serious^e^	Publication bias strongly suspected^c^	⨁◯◯◯ Very low	22/564 (3.9%)	26/556 (4.7%)	RR: 1.19 (0.69 to 2.05)	22/564 (3.9%)	7 more per 1000 (from 12 fewer to 41 more)
Total hospital stay											
1321 (3 RCTs)	Very serious^a,b^	Not serious	Not serious	Not serious	Publication bias strongly suspected^c^	⨁◯◯◯ Very low	670	651	-	670	MD: 1.96 lower (2.55 lower to 1.37 lower)
SCBU admission											
1643 (4 RCTs)	Very serious^a,b^	Not serious	Serious^f^	Serious^e^	Publication bias strongly suspected^c^	⨁◯◯◯ Very low	21/830 (2.5%)	23/813 (2.8%)	RR: 1.17 (0.65 to 2.10)	21/830 (2.5%)	4 more per 1000 (from 9 fewer to 28 more)

Comparison of outpatient induction with Foley catheter versus inpatient induction with prostaglandins: The interval from induction to delivery interval was observed to have had a little increase in outpatient IOL with Foley catheter vs. inpatient with prostaglandins, (MD: 1.1 higher, 1.77 lower to 3.98 higher). No difference was observed in the cesarean section rate (549, 2 RCTs, RR: 1.28, 95% CI: 0.96 to 1.71), and SCBU admission (549, 2 RCTs, RR: 0.80, 95% CI: 0.56 to 1.16), as shown in Table 7.

**Table 7:** Subgroup analysis: outpatient Foley catheter vs. inpatient hormonal (prostaglandins) induction of labor CI: confidence interval; MD: mean difference; RR: risk ratio

Analysis or subgroup title	No. of studies	No. of participants	Statistical method	Effect size
Cesarean section rate	2	549	RR (IV, fixed, 95% CI)	1.28 [0.96, 1.71]
Special care baby unit admission	2	549	RR (IV, fixed, 95% CI)	0.80 [0.56, 1.16]
Induction to delivery interval	2	549	MD (IV, fixed, 95% CI)	1.10 [-1.77, 3.98]

**Figure 17:**
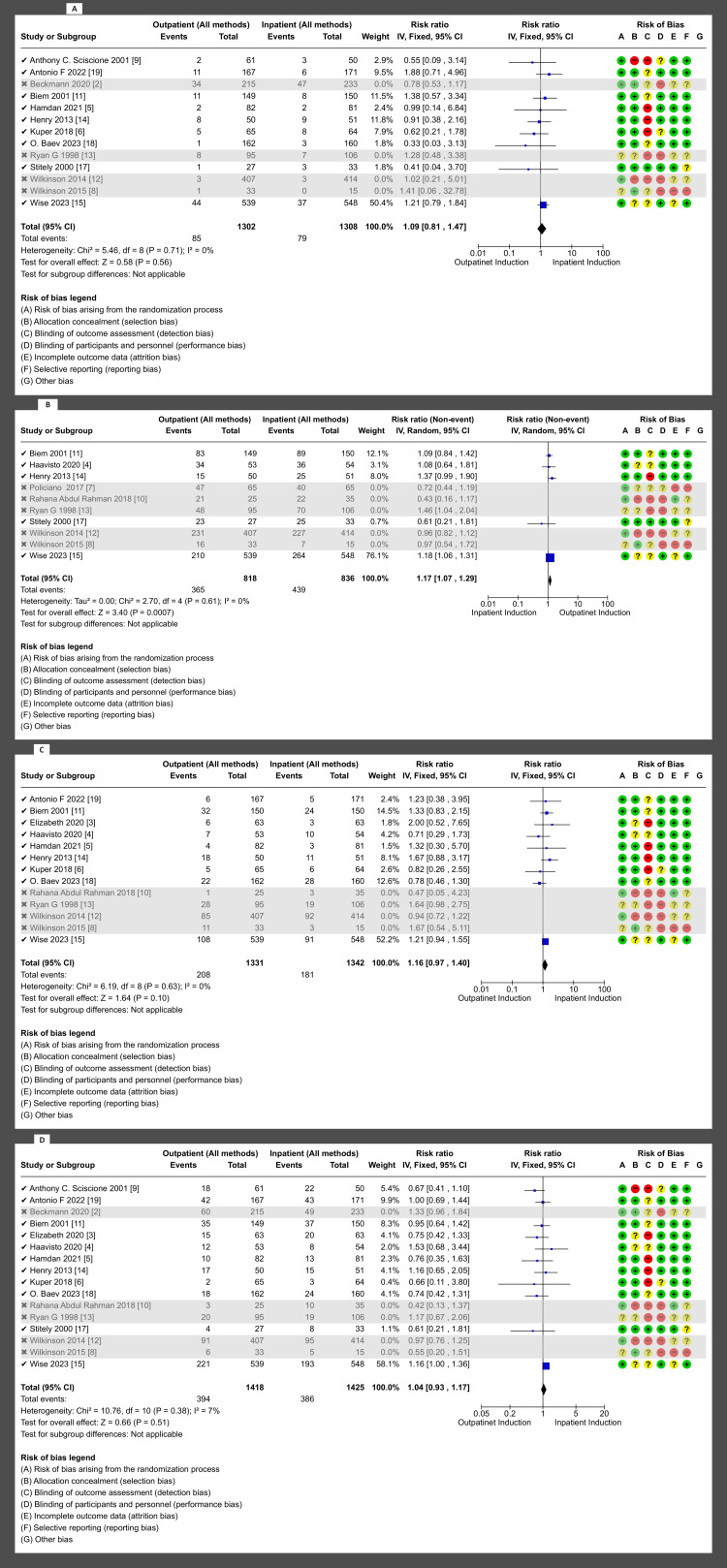
Senstivity analysis of primary outcome in the main analysis A: Cesarean section rate B: Operative vaginal delivery C: Vaginal delivery rate D: Special care baby unit admission

Sensitivity analysis (Figure 17) was conducted for the primary outcomes, excluding studies with a risk of bias. The analysis revealed no difference in the outcomes after excluding the studies with a high risk of bias and borderline results.

Discussion

In this review, 18 RCTs were included, involving 4886 patients, to compare IOL in outpatient and inpatient settings using different methods. As expected, there was clinical heterogeneity between the selected studies, and hence subgroup analysis was performed to compare the same induction methods used in outpatient versus inpatient settings. Regarding the main outcomes, there was no difference in the rates of cesarean section, operative vaginal delivery, and vaginal delivery between outpatient and inpatient induction, regardless of the method used. No differences were observed in other perinatal, labor and delivery, or neonatal outcomes. However, it was noted that outpatient induction resulted in a shorter interval from induction to the onset of labor, as well as a shorter interval from admission to delivery. Additionally, the outpatient group had a shorter hospital stay.

These findings suggested that outpatient induction is a safe option for reducing hospital stays in low-risk women undergoing induction of labor. Of note, no major morbidities were mentioned in the included studies, and there was no difference in febrile morbidities between the outpatient and inpatient groups. Dong et al. reported similar findings: no difference in perinatal, labor, and neonatal outcomes, as well as shorter hospital stays with outpatient induction compared to inpatient induction, in their systematic reviews [1,41,42,43]. They concluded that outpatient induction can be safely conducted in selected populations. Furthermore, when comparing outpatient and inpatient induction with a Foley catheter, a reduced rate of cesarean section, shorter intervals from induction to delivery, and decreased total hospital stay were observed (low certainty of evidence) without affecting labor or neonatal outcomes [1,44,45].

When comparing inpatient induction with prostaglandin to outpatient induction, subgroup analysis revealed no significant differences in reported outcomes (very low certainty of evidence). The only notable difference was in hospital stay duration, which was shorter for the outpatient group. Zarco et al. reported similar findings [41], as did Mazzoli et al. in their systematic review [42]. However, Mazzoli et al. noted a slight increase in rates of uterine hyperstimulation in the outpatient group, unlike our findings. We attribute this discrepancy to the low number and quality of studies included, which were insufficient to draw conclusions. Also, Mazzoli et al. included two out of three studies with retrospective cohorts, and the overall quality of the studies was too weak to reach a definitive conclusion. In the last subgroup analysis between outpatient Foley catheter induction and inpatient induction with hormonal methods, no difference was demonstrated between the methods in cesarean section rate and SCBU admission; however, the interval from induction to delivery slightly increased in the outpatient Foley catheter group.

We recommend further studies comparing outpatient induction with various methods, as there is a lack of research in this area. Alfirevic et al. [41] have suggested using cohort study designs for this comparison, as it would provide more information about patient perspective and effectiveness compared to randomized trials where blinding is not possible, which lowers the strength of evidence.

Limitations of the Evidence Included in the Review

The analysis included numerous RCTs, but there were limitations noted, including significant variability between the studies in the reported outcomes. Additionally, most of the included RCTs were open-label, and hence blinding was not possible. However, blinding of outcome assessors should be attempted at least to reduce the risk of bias. Most of the evidence was downgraded due to the risk of bias on blinding with the GRADE approach. Although it was one of the secondary outcomes included in the protocol, patient satisfaction was not included in the outcomes due to significant variations in methods used to assess it and the short time interval from delivery. We recommend a longitudinal study design to measure maternal satisfaction during the induction process, at three, six, and 12 months post-induction, to gain a better understanding of women's level of satisfaction with outpatient induction.

However, despite these limitations, we recommend that outpatient induction in selected low-risk pregnant populations should be implemented. The choice of methods should depend on local protocol, the feasibility of follow-up, and the accessibility of the hospital.

## Conclusions

In this meta-analysis, outpatient IOL was found to have no significant differences in terms of main labor (low to very low certainty of evidence), delivery (moderate to low level of evidence), and neonatal outcomes (moderate level of evidence) vs. inpatient IOL. Furthermore, subgroup analysis of Foley catheter induction as an outpatient showed a little reduction in cesarean section rate (low certainty of evidence) with no reported morbidities; hence, outpatient induction has been proven to be as safe and equally effective as inpatient induction. These results are most applicable in well-developed settings with the feasibility of follow-up and quick intervention, with adequate awareness among women. Moreover, these findings apply to low-risk pregnant women. High-risk pregnant populations generally undergo induction as inpatients to enable timely intervention in cases of maternal or fetal compromise. Outpatient induction for high-risk women has not been extensively studied and may be considered unethical. Further research on outpatient IOL with various agents is recommended to test the efficacy of all methods in an outpatient setting.
